# Measuring trends in extinction risk: a review of two decades of development and application of the Red List Index

**DOI:** 10.1098/rstb.2023.0206

**Published:** 2025-01-09

**Authors:** Stuart H. M. Butchart, H. Resit Akçakaya, Alex J. Berryman, Thomas M. Brooks, Ian J. Burfield, Janice Chanson, Maria P. Dias, John S. Donaldson, Claudia Hermes, Craig Hilton-Taylor, Mike Hoffmann, Jennifer A. Luedtke, Rob Martin, Amy McDougall, Kelsey Neam, Beth Polidoro, Domitilla Raimondo, Ana S. L. Rodrigues, Carlo Rondinini, Claire Rutherford, Tom Scott, Ashley T. Simkins, Simon N. Stuart, Jemma Vine

**Affiliations:** ^1^ BirdLife International, David Attenborough Building, Pembroke Street, Cambridge CB2 3QZ, UK; ^2^ Department of Zoology, University of Cambridge, Downing Street, Cambridge CB2 3EJ, UK; ^3^ Department of Ecology and Evolution, Stony Brook University, New York, NY 11794-5245, USA; ^4^ International Union for Conservation of Nature Species Survival Commission, Rue Mauverney 28, Gland 1196, Switzerland; ^5^ International Union for Conservation of Nature, Rue Mauverney 28, Gland 1196, Switzerland; ^6^ Re:wild, PO Box 129, Austin TX 78767, USA; ^7^ IUCN SSC Amphibian Specialist Group, IUCN, Rue Mauverney 28, Gland 1196, Switzerland; ^8^ Department of Animal Biology, Centre for Ecology, Evolution and Environmental Changes & CHANGE - Global Change and Sustainability Institute, Faculty of Sciences of the University of Lisbon, Campo Grande, Lisboa 1749-016, Portugal; ^9^ Department of Plant and Soil Sciences, University of Pretoria, Private Bag X20, Hatfield, 0028, South Africa; ^10^ South African National Biodiversity Institute, Private Bag X101, Pretoria 0001, South Africa; ^11^ International Union for Conservation of Nature Red List Unit, David Attenborough Building, Pembroke Street, Cambridge CB2 83QZ, UK; ^12^ Zoological Society of London, Regent's Park, London NW1 4RY, UK; ^13^ School of Mathematical and Natural Sciences, Arizona State University, Glendale AZ 85306, USA; ^14^ CEFE, University of Montpellier, CNRS, EPHE, IRD, Montpellier, France; ^15^ Global Mammal Assessment Program, Department of Biology and Biotechnologies, Sapienza University of Rome, Viale dell’Università 32, Rome 00185, Italy; ^16^ Synchronicity Earth, 1 Chancery Lane, London WC2A 1LF, UK; ^17^ A Rocha International, 180 Piccadilly, London W1J 9HF, UK

**Keywords:** IUCN Red List, extinction risk, biodiversity conservation, multilateral environmental agreement, indicator

## Abstract

The Red List Index (RLI) is an indicator of the average extinction risk of groups of species and reflects trends in this through time. It is calculated from the number of species in each category on the IUCN Red List of Threatened Species, with trends influenced by the number moving between categories when reassessed owing to genuine improvement or deterioration in status. The global RLI is aggregated across multiple taxonomic groups and can be disaggregated to show trends for subsets of species (e.g. migratory species), or driven by particular factors (e.g. international trade). National RLIs have been generated through either repeated assessments of national extinction risk in each country or through disaggregating the global index and weighting each species by the proportion of its range in each country. The RLI has achieved wide policy uptake, including by the Convention on Biological Diversity and the United Nations Sustainable Development Goals. Future priorities include expanding its taxonomic coverage, applying the RLI to the goals and targets of the Kunming–Montreal Global Biodiversity Framework, incorporating uncertainty in the underlying Red List assessments, integrating into national RLIs the impact of a country on species’ extinction risk abroad, and improving analysis of the factors driving trends.

This article is part of the discussion theme issue ‘Bending the curve towards nature recovery: building on Georgina Mace's legacy for a biodiverse future’.

## 1. Introduction

Human impacts on the planet are driving species extinct, with loss of biodiversity reducing the vital benefits that people receive from nature and threatening the quality of life of current and future generations [1]. Responding to this biodiversity crisis requires an understanding of how many and which species are being driven closer to extinction, the rate of extinctions and trends in extinction risk, as well as where to focus conservation efforts, which threats need to be mitigated, and whether interventions are successful.

The International Union for Conservation of Nature (IUCN) Red List of Threatened Species (hereafter ‘the IUCN Red List’) was developed to meet these needs. Its origins can be traced back to 1933 when John Charles Phillips suggested the need to survey and document the status of extinct and threatened species [2], prompting the production of the first such lists for mammals [3,4] and birds [5,6]. These evolved into a card index by 1959, and data sheets on ‘Animals and Plants Threatened with Extinction’ by 1962 [7]. In 1964, lists of rare birds and mammals were published as supplements to the IUCN Bulletin [8,9]. Later in the 1960s, a series of volumes of loose-leaf data sheets in red binders (termed ‘Red Data Books’ by Sir Peter Scott) were produced for mammals, birds, amphibians, reptiles, fishes and flowering plants (e.g. [10]). Through the late 1970s and 1980s, a series of properly bound Red Data Books were published by IUCN (e.g. [11]) and the International Council for Bird Preservation (ICBP, now BirdLife International; e.g. [12]). In 1986, the first ‘IUCN Red List of Threatened Animals’ was published [13] with minimal information on each species, followed much later by an analogous list for plants [14]. The first comprehensive assessment covering all threatened species in any group was published for birds in 1988 [15].

All these earlier publications used categories of extinction risk with rather broad and subjective definitions, leading to inconsistencies and challenges in their application. Following some unsuccessful attempts to address these problems (e.g. [16]), the IUCN Species Survival Commission Steering Committee appointed Georgina Mace in 1988 to lead the development of a new system of Red List Categories and Criteria. The first proposals for a revised approach were drafted in 1989 and published in 1991 [17]. After further discussions, a revised proposal [18] underwent extensive review, testing (on over 2000 species across the taxonomic spectrum) and revision [19]. The new system was adopted in 1994 [20], with minor revisions in 2001 [21].

The revised criteria were based on principles of population biology and aimed to reflect the main symptoms of extinction risk, be applicable to a wide range of taxa and take into consideration uncertainty and lack of data [22]. The five criteria relate to parameters including the rate of population reduction, population size and structure, and geographic range and decline, with different quantitative thresholds for each extinction risk category [21]. The criteria are used to assign species to different categories of extinction risk: Least Concern, Near Threatened, Vulnerable, Endangered, Critically Endangered, Extinct in the Wild, and Extinct, in increasing order of risk. Species in the Vulnerable, Endangered and Critically Endangered categories are collectively referred to as ‘threatened’. Species are classified as Data Deficient if there is insufficient information to apply the criteria [21].

These new criteria and categories were applied for the first time to all birds in 1994 [23], and to all mammals in 1996 [24]. Over 166 000 species have now been globally assessed and published on the IUCN Red List across a wide range of taxonomic groups, including vertebrates, invertebrates, plants, fungi and protists; with over 46 000 species listed as threatened [25]. Georgina Mace played a pivotal role throughout the process of developing and applying the Red List criteria, bringing the rigour of population biology to the practical challenges of assessing extinction risk consistently across different groups of species and types of life histories. She also played an important role in promoting the taxonomic expansion of the Red List, strengthening the requirements for documenting assessments and contributing to the development of the Red List Index (RLI [26]).

The IUCN Red List is now widely regarded as the most objective system for classifying species’ extinction risk [27,28], informing policy and practice worldwide. While the name ‘Red List’ was inherited from its inception as a list of species of conservation concern, today it is progressively being applied to evaluate the extinction risk of entire taxonomic groups, including threatened as well as non-threatened species. Furthermore, it has evolved from a list into a comprehensive database compiling information on the global extinction risk and conservation of species, including detailed text accounts, quantitative tabular data (on population size, trends, range size, number of subpopulations and other relevant parameters) and systematic documentation of ecology, threats, conservation actions, habitats and utilization for each species [25]. The majority of assessments appearing on the IUCN Red List are carried out through the auspices of the Red List Partnership, involving members of the IUCN Species Survival Commission (typically organized into Specialist Groups, e.g. Amphibian Specialist Group, and appointed Red List Authorities, e.g. BirdLife International), Red List Partner institutions (e.g. Re:wild, Zoological Society of London) or specialists working on IUCN-led assessment projects.

Data from the IUCN Red List are used to calculate the RLI, a measure that reflects the level and trend in extinction risk for sets of species. Here, we review developments and applications of the RLI over the two decades since its publication [26] and identify priorities for the future.

## 2. Methodological development of the RLI

The development of the RLI was originally motivated by BirdLife International’s desire to compare its first three comprehensive assessments of the extinction risk of the world’s birds [15,23,29] and to assess overall trends in their status, given the lack of global biodiversity indicators for assessing progress towards a target adopted by the Convention on Biological Diversity (CBD) to significantly reduce the rate of loss of biodiversity by 2010 [30].

While the availability of repeated comprehensive assessments for birds provided the possibility of tracking trends in the global extinction risk of species over time, it was clear that simple comparisons of the lists (e.g. using the number of threatened species) would be misleading because most changes resulted from improved knowledge, revised taxonomy, changes to the Red List criteria, or other reasons unrelated to extinction risk. It was therefore necessary to identify the species that had changed Red List categories across the three assessments, and determine which changes resulted from a genuine improvement or deterioration in extinction risk [26]. A method was then developed for generating a biodiversity indicator from these data, which was termed the RLI, with two key innovations. First, the method involved retrospectively reassessing the extinction risk of species at earlier time-points based on the most recent knowledge. Second, it considered changes in different levels of extinction risk, not simply the proportion of species that are threatened [26].

The RLI was designed as a measure of aggregated likelihood of species' survival, such that it declines as species’ status deteriorates (instead of being an index of extinction risk that increases as risk increases). This makes it intuitive to interpret alongside other metrics of the state of biodiversity that decline as the state deteriorates, like the extent of natural ecosystems or metrics of population abundance.

### (a) Original method and its shortcomings

The original method for calculating the RLI involved multiplying the number of species in each Red List category by a weight and summing these products to give a total score, as in equation (2.1). Between assessment periods, the net number of genuine changes to the total in each category was calculated, multiplied by the category weight and summed to calculate the proportional change in the total score (equation (2.2)). The value of the index was set to 100 in the first assessment year. For subsequent assessments, the index value was then calculated by multiplying the proportional change by the previous index value (equation (2.3)) [26,31]. Specifically, where *T* is total score, *N_c_(t_i_*) is the number of species in category *c* at time *t_i_*, where *t_i_* is the year of the *i*th assessment (assessments are not necessarily made every year); *W_c_* is the weight for category *c*; *P* is proportional genuine change; *I_t_i* is the value of the index at time *t_i_*; *c*(*t_i_*, *s*) is the category of species *s* at time *t_i_*; *W_c_* is the weight for category *c*; *G_s_* = 1 if change (from *t*_(*i *− 1)_ to *t_i_*) in category of species *s* is genuine (otherwise *G_s_* = 0) and where *I_t_i *− *1* = 100 for the first year of assessment:


(2.1) $$T_{t_{i}}=\sum_{c}W_{c}.N-c_{\left(t_{i}\right)},$$

(2.2) $$P_{t_{i}}=\sum_{s}\left[\left(W_{c\left(t_{i},s\right)}-W_{c\left(t_{i-1},s\right)}\right)\cdot G_{s}\right]/T_{t_{i-1}}$$

(2.3) $$I_{t_{i}}=I_{\left(t_{i-1}\right)}.\left(1-P_{t_{i}}\right).$$

The simplest way of setting category weights is using an ‘equal steps’ approach, where Least Concern = 0, Near Threatened = 1, Vulnerable = 2, Endangered = 3, Critically Endangered = 4 and Extinct = 5. This reflects the fact that the Red List categories are ordinal ranks in extinction risk. However, the steps between lower categories translate to smaller increases in extinction risk than steps between higher categories. Therefore, the RLI was also calculated using weights based on the relative extinction risk associated with each category (derived from the quantitative thresholds specified in each criterion), where Least Concern = 0, Near Threatened = 0.0005, Vulnerable = 0.005, Endangered = 0.05, Critically Endangered = 0.5 and Extinct = 1 [26]. The latter approach produces an index that is largely influenced by movements of species in or out of the Critically Endangered category. Hence, subsequent RLIs have mainly been calculated using the equal steps approach to also reflect changes in the extinction risk of less-threatened species.

The original RLI formula was developed and tested using data for birds, showing a 7% decline in the index from 1988 to 2004 [26]. However, the application of this method to amphibian data [31] and subsequent testing identified three drawbacks to this initial mathematical formulation. First, the RLI performed inappropriately once it had reached zero. Indeed, if the RLI value for a set of species declined by 100% (to 0), i.e. if the average threat score was double that of the previous assessment, the RLI could not subsequently change. If the RLI value decreased below zero, further deterioration in the status of the set of species counterintuitively increased the RLI value. Second, RLI values were affected by the frequency of assessments, which differs between taxonomic groups: the RLI value at a particular time point was dependent on the number of assessments since the first because it was calculated in relation to the value for the previous assessment. Third, newly evaluated species could introduce bias. These included species that were newly recognized taxonomically, or that were previously assessed as Data Deficient, which contributed to the index value only from the point when they were assessed for the second time. If the extinction risk of such newly added species changed at a different average rate from the original set, this affected the RLI trend.

### (b) Revised method

To address these shortcomings of the original method for calculating the RLI, a revised method was developed [32] in which the number of species in each category is multiplied by the category weight, and summed across all categories. This total is divided by the product of the total number of species and the maximum weight, and subtracted from 1. Mathematically this can be expressed through equation (2.4), where RLI*_t_* = the index value at time *t*; *W_c_*_(_*_t_*_,_*_s_*_)_ *=* the weight for Red List category *c*, which is summed for all species *s* at time *t*; *W*_EX_ = the weight for the Extinct category; and *N* is the total number of assessed species, excluding those considered Data Deficient:


(2.4) $${RLI}_{t}=1-\frac{\sum_{s}W_{c\left(t,s\right)}}{W_{EX}\cdot N}.$$

This method requires that the same set of species be included in all time steps, and that the only category changes are those resulting from genuine improvement or deterioration in extinction risk. This condition can be met by ‘back-casting’, a process of retrospectively adjusting earlier Red List categories using current information and taxonomy, and assuming that the current Red List category has applied since the species was first assessed, unless information suggests that a genuine change has occurred (e.g. [33]).

The revised approach results in an index ranging from 1 (all species are Least Concern) to zero (all species are Extinct). Hence the resulting index illustrates both the overall relative extinction risk aggregated across species at any given point in time (the value of the RLI) and its trend (the gradient of the line). Trends in the index represent the balance between deteriorations in species’ status (i.e. increases in extinction risk) and improvements in status (i.e. reductions in extinction risk), including those resulting from successful conservation action. A negative RLI trend means that the expected rate of future species' extinctions is growing (i.e. the rate of biodiversity loss is increasing). A positive trend means that the expected rate of species' extinctions is reducing (i.e. the rate of biodiversity loss is decreasing) and a horizontal line means that the expected rate of species' extinctions is not changing (i.e. biodiversity loss is continuing) [31].

### (c) Aggregating indices and calculating confidence intervals

Following the first calculation of an RLI for birds, multiple comprehensive Red List assessments have allowed RLIs showing trends over time to be calculated for amphibians, mammals, cycads and warm-water reef-building corals (with baseline data points calculated for several other groups). RLIs are typically calculated only for groups in which all species have been assessed (or for randomized samples of species: see below) to avoid bias. An aggregated RLI has been developed by combining data from multiple taxonomic groups [34]. This is achieved by interpolating between RLI values for each group, extrapolating to extend trends when assessments are asynchronous, and calculating the aggregated trend as the mean across constituent taxon RLIs.

Confidence intervals are generated by modelling the RLI for each taxonomic group to account for various sources of uncertainty. (i) Data Deficiency: Red List categories (from Least Concern to Extinct) are assigned to all Data Deficient species with a probability proportional to the number of species in each non-Data Deficient category for that taxonomic group. (ii) Extrapolation uncertainty: although RLIs are extrapolated linearly based on the slope of the closest two data points, there is uncertainty about how accurate this slope may be, so rather than extrapolating deterministically, the slope is selected from a normal distribution with a probability equal to the slope of the closest two data points, and standard deviation equal to 60% of this slope. (iii) Temporal variability: because assessments are repeated only at multi-year intervals, the precise value for any particular year is uncertain, so the RLI value for a given taxonomic group in a given year is assigned from a moving window of 5 years, centred on the focal year (with the window set as 3−4 years for the first 2 and last 2 years in the series). Hence, to incorporate these uncertainties into the aggregated RLI, Data Deficient species are allotted a category as described above, an RLI for each taxonomic group is calculated interpolating and extrapolating as described above, and a final RLI value is assigned to each taxonomic group for each year from a window of years as described above. This is repeated (i.e. bootstrapped) 10 000 times for each taxonomic group, and the mean is calculated [34].

### (d) RLIs based on a sampled Red List approach

For very speciose and/or poorly known taxonomic groups, comprehensive assessments of all species are unfeasible in the near future. To address this, a sampled approach to Red Listing was developed, enabling the generation of RLIs for sampled groups [35]. Data from repeated Red List assessments for birds were used to investigate how large a sample of species is required to generate a robust trend, showing that a random sample of c. 900 species is sufficient if the group changes in extinction risk at the same rate as birds [35]. Subsequent work using an enlarged dataset showed that a sample of 200−400 species may be sufficient to detect whether the trend in extinction risk is increasing or decreasing overall if reassessments are conducted every 10 years, but correctly detecting changes in slope (e.g. an accelerating increase or decrease in extinction risk) requires samples of ≥900 species [36].

Application of the sampled Red List approach has generated baseline RLI data points for several less comprehensively assessed taxonomic groups, including freshwater crabs [37], dragonflies and damselflies [38], and plants (based on bryophytes, pteridophytes, gymnosperms, monocotyledons and legumes [39]), with work ongoing to reassess these groups and calculate RLI trends for the sampled sets of species.

## 3. Policy applications of the RLI

**Figure 1.**
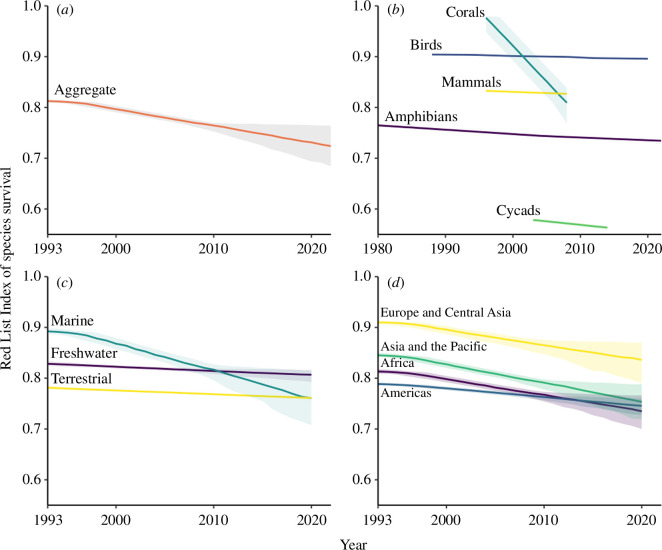
Red List Indices of species survival (*a*) aggregated across five groups: mammals (5899 species), birds (11 147), amphibians (8011), warm-water reef-building corals (859) and cycads (340); (*b*) for each of these taxonomic groups individually; (*c*) disaggregated by realm: terrestrial (23 490 species), freshwater (7032), marine (1840); and (*d*) disaggregated by region: Africa (5704 species), Americas (10 703), Asia and the Pacific (9707) and Europe and Central Asia (2040). Shading shows confidence intervals.

**Figure 2.**
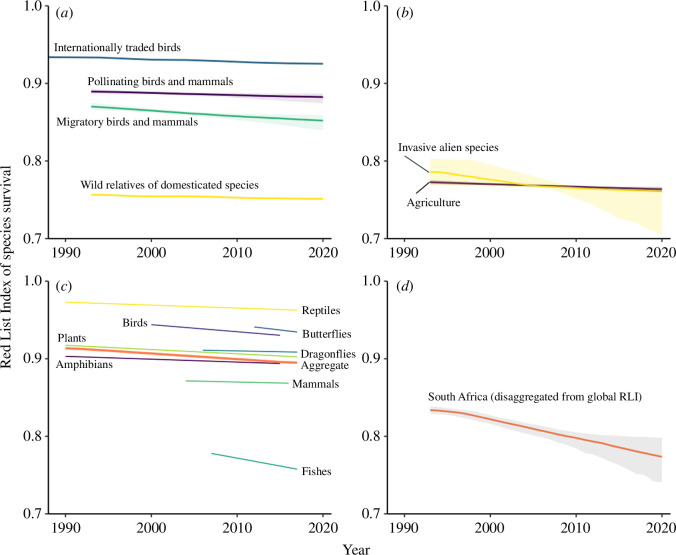
Red List Indices of species survival for: (*a*) bird species recorded in international trade (4318 species), bird (1170) and mammal species (367) known to pollinate plants, migratory bird (2199) and mammal (205) species, and bird (240) and mammal species (58) that are wild relatives of farmed and domesticated species; (*b*) mammals (5899 species), birds (11 147), amphibians (8011), warm-water reef-building corals (859) and cycads (340), showing trends driven by agriculture or invasive alien species; (*c*) South Africa, based on repeated assessments of national extinction risk for birds (732 species), mammals (336), amphibians (126), reptiles (397), freshwater fishes (118), dragonflies (163), butterflies (799), a sample of plants (900) and an aggregated index across all these groups (3571); and (*d*) South Africa, showing the global RLI disaggregated for this country, with each species (312 mammals, 751 birds, 126 amphibians, 97 corals and 38 cycads) weighted by the proportion of its global range within the country. Shading shows confidence intervals.

The global RLI based on data aggregated across mammals, birds, amphibians, corals and cycads has had wide global and regional policy uptake, and a number of disaggregations have been developed to address different policy needs, including the biodiversity goals, targets and objectives relating to various multilateral environmental agreements, and global and regional environmental reporting processes. These are reviewed below, with the different policy applications of the RLI illustrated by graphs for several versions of the indicator (figures 1 and 2; see electronic supplementary materials for the relevant data, sources, and numbers of species).

### (a) Intergovernmental conventions and environmental reporting

The RLI and various disaggregations have been widely used by intergovernmental conventions and global environmental reporting processes [40]. For example, the RLI is used to track progress towards the United Nations Sustainable Development Goal (SDG) 15 (‘Life on Land’), specifically target 15.5 (on action to halt the loss of biodiversity [41]). The RLI was also used alongside other indicators in the various editions of the CBD’s Global Biodiversity Outlook to demonstrate that the world failed to meet both the ‘2010 target’ to significantly reduce the rate of loss of biodiversity, and Aichi Target 12 to prevent extinctions by 2020 [42,43]. It has been adopted as a ‘headline indicator’ for Goal A (which commits CBD Parties to reducing the extinction rate and risk for all species tenfold by 2050) and Target 4 (on urgent management actions for threatened species) of the Convention’s Kunming–Montreal Global Biodiversity Framework [44]. Disaggregations of the RLI are also relevant to Goal B and target 5 (on sustainable use of biodiversity), target 2 (on restoration), target 6 (impacts of invasive alien species), target 7 (impacts of pollution), target 9 (benefits from the use of biodiversity), target 10 (sustainable management of agriculture, aquaculture, fisheries and forestry) and target 11 (nature’s contributions to people).

Other multilateral environmental agreements that use the RLI for tracking progress towards their strategic objectives include the Convention on the Conservation of Migratory Species of Wild Animals (CMS) [45,46] and some of its daughter agreements (e.g. [47,48]), as well as the Convention on Wetlands of International Importance especially as Waterfowl Habitat (the ‘Ramsar Convention’) [49] and the Convention to Combat Desertification [50]. The RLI has been widely used in reports on the state of the environment, such as the first Global Assessment of the Intergovernmental Science-Policy Platform on Biodiversity and Ecosystem Services (IPBES) [51], as well as several regional (e.g. for Africa [52]) and thematic (e.g. pollination [53]) assessments. It has also been profiled in editions of the Global Environment Outlook (e.g. [54]) and the Living Planet Report (e.g. [55]).

### (b) RLIs for different subsets of species of policy relevance

RLIs have been calculated for species in different ecosystems (e.g, figure 1*c*), showing that marine species are declining at a faster rate than terrestrial or freshwater species. Similarly, disaggregations are available (on the IUCN Red List website at https://www.iucnredlist.org/search) for forest-specialist and wetland-specialist species, showing that across all birds, mammals, amphibians and cycads, forest species are more threatened (i.e. with lower RLI values) than wetland species. Trends in wetland species are highly relevant to the Ramsar Convention, which aims to conserve the habitats of such species.

Regional RLIs aggregated from the five comprehensively assessed groups show that species in all regions are deteriorating in status (i.e. RLI values are declining; figure 1*d*). Other disaggregations of the RLI show that migratory species of birds and mammals are declining (figure 2*a*), but are less threatened overall than non-migratory species [46], reflecting their generally larger distributions and populations, despite rapid and chronic declines in many migratory species (e.g. [56]). This measure is used by the CMS as an overall indicator of the health of those species that the convention aims to conserve [45,46]. Similarly, bird and mammal species documented as plant pollinators (such as hummingbirds and fruit bats) are deteriorating in status, with potential impacts on ecosystem functions and services [57] (figure 2*a*). This metric was used for assessing progress towards Aichi Target 14 on the restoration and safeguarding of ecosystems that provide essential services [43,58].

Wild relatives of domesticated bird and mammal species have a declining RLI trend, indicating that these related species are increasingly being driven towards extinction. This risks potential loss of genetic diversity, reducing the potential development of more productive, nutritious and resilient breeds in future, and hence risking future food security [59] (figure 2*a*). This metric was used for assessing progress towards Aichi Target 13 on maintaining the genetic diversity of cultivated plants and domesticated animals and their wild relatives [43,58]. More generally, RLIs for utilized species (i.e. those used for food and medicine, and internationally traded species) all show negative trends, with implications for a range of ecosystem services [43,58,60].

### (c) RLIs showing impacts of particular drivers

The status of some groups of species is so closely linked to particular drivers that they can be used to measure the impacts of policies directly. For example, RLIs for tunas, billfishes and sharks for 1950−2019 (with annual values calculated using a Bayesian framework to model population time series and estimate probabilistic extinction risk applying Red List criterion A) show that after at least half a century of increasing extinction risk, effective fisheries management has reversed declines in the RLIs for tunas and billfishes, whereas the RLI for sharks, which are inadequately managed, continues to decline [61].

Other RLIs for particular subsets of species are more difficult to interpret in a particular policy context. For example, the RLI for internationally traded species is relevant to the Convention on International Trade in Endangered Species of Wild Fauna and Flora (CITES) [60] (figure 2*a*), but changes in the status of such species are driven by many factors, not just international trade (e.g. many traded parrot species are also heavily impacted by habitat loss). An alternative and more pertinent approach in such contexts is to disaggregate the RLI to show trends in extinction risk for all species (not just a particular subset), but to include genuine changes in status driven only by a particular threat (or its successful mitigation). Data associated with each genuine Red List category change have been used to identify its primary driver (i.e. the threat(s) causing the change in status, or the threat(s) mitigated through conservation action), allowing the production of a range of RLIs showing trends driven by particular threats, such as agriculture or invasive alien species (e.g. [62], figure 2*b*), which are used in different policy fora (e.g. [44]). However, these RLIs do not currently reflect the impacts of the threat on species for which it is not the primary driver of a change in status: further development of methods to address this, as well as improved methods for attributing changes in status to drivers, are future priorities (see below).

### (d) National and regional RLIs

RLIs can also be calculated for individual countries, and this has been done in two ways [63,64]. First, the global RLI can be disaggregated for each country, weighting each species by the proportion of its global range within the country, such that the indicator illustrates the aggregate extinction risk for species within the country relative to its potential contribution to global species' extinction risk, within the taxonomic groups included [65]. This provides an index that is comparable for all countries worldwide, but that may be more informative for countries with higher levels of endemism, for which changes in the size and trends of the population and distribution of the species within the country have a greater influence on the species’ global Red List category. An example for South Africa is shown in figure 2*d*, while disaggregated RLIs for all countries are provided on the IUCN Red List website (https://www.iucnredlist.org/search), Target Tracker platform (https://target-tracker.org/), Integrated Biodiversity Assessment Tool (https://www.ibat-alliance.org/country_profiles) and the SDGs indicators database (https://unstats.un.org/sdgs/indicators/database/).

The second approach is through national RLIs based on data from repeated assessments of extinction risk at the national scale for national red lists, typically applying the Guidelines for Application of the IUCN Criteria at Regional and National Levels [66] (e.g. [63,67–71]; figure 2*c*). These show trends in national-level extinction risk for the species groups included (which in some cases extend beyond the five currently included in national disaggregations of the global RLI). The two approaches are complementary, with the disaggregated global RLIs showing national contributions toward global extinction risk trends, whereas the national RLIs show trends in national-level extinction risk [63].

During 2010−2020, only six nations included national RLIs in their Sixth National Reports to the CBD. This may be because the list of indicators recommended by CBD (including the RLI) was only published in 2016, and/or countries may have preferred to focus on their nationally generated indicators rather than those suggested by CBD [63]. Alternatively, the low uptake may be because of challenges in applying the Red List criteria and RLI at the national scale, e.g. owing to a degree of subjectivity in treatment of extralimital populations on the likelihood of national extinction [72], substantial variation between species groups in national extinction risk levels and trends making it difficult to interpret an aggregated index [73], the need for sufficient numbers of species and species groups to produce a representative national index [63,74], and the costs and time to achieve this [63]. Long intervals between reassessments of taxa for national Red Lists owing to lack of resources may pose a particular challenge. National-scale extinction risk is also probably more meaningful to consider in larger countries with more endemic species.

New National Report templates for the Kunming–Montreal Global Biodiversity Framework will be pre-populated with national values (where available) for all countries, for the ‘headline indicators’ (including the RLI) adopted by the CBD; countries can then choose to replace these with their own nationally generated indicators if these exist. This, and the fact that many nations have national Red Lists [63], is likely to lead to an increase in the number of countries using this metric in their national reports. As noted below, increasing the number of taxonomic groups assessed and the frequency with which they are reassessed is important in order to ensure that national RLIs and national disaggregations of the global RLI are meaningful.

The same two country-level approaches can be applied to other policy-relevant spatial units. For example, regional RLIs disaggregated from the global index are available for all regions following the different regional classifications used by the SDGs, IPBES and CMS [75] (see also https://www.iucnredlist.org), while the second approach of assessing extinction risk at the regional scale has been applied in the European Union to generate regional RLIs for multiple taxonomic groups [76].

### (e) Measuring conservation impact

The RLI has also been used to quantify conservation impact, comparing two versions of the RLI for terrestrial vertebrates: one with the observed changes and a counterfactual excluding improvements in status (i.e. cases where a species moved to a Red List category of lower extinction risk) owing to conservation action [77]. This found that 7% of genuine category changes (68/928 during 1980−2008) represented improvements in the status of species, and all but four of these resulted from conservation action. This means that the rate of deterioration of terrestrial vertebrates would have been at least one-fifth greater in the absence of conservation action. For birds, conservation action reduced the decline in the RLI between 1988 and 2008 from 0.58% to 0.49%, equivalent to preventing 39 species each moving one Red List category closer to extinction. For mammals, conservation action reduced the RLI decline between 1996 and 2008 from 0.94% to 0.8%, equivalent to preventing 29 species from moving one category closer to extinction. Furthermore, these results underestimated the impact of conservation because they did not account for species that either would have deteriorated further in the absence of conservation actions or that improved in status, but not enough to qualify for a lower Red List category [77]. Subsequent (albeit more narrowly focussed) studies suggest that the magnitude of this underestimate may be considerable (e.g. [33,67]).

This approach was further developed to calculate the conservation impact of an organization, the Durrell Wildlife Conservation Trust [78]. A counterfactual RLI was estimated for 1988−2012 in the absence of conservation for 17 vertebrate species targeted for action by the Trust. The counterfactual RLI was calculated not just by focussing on species that changed Red List category, but by predicting the category under which each of the species would have qualified in 2012 if all conservation actions led or supported by the institution had ceased in 1988 and if the threats at the time had continued unabated. The results show the striking impact of conservation action on the extinction risk of this small set of species: while the actual RLI increased by 67% (because many of the species improved in status owing to conservation efforts), the counterfactual approach showed that the RLI would have declined by 23% in the absence of conservation action [78]. This counterfactual approach has also now been extended to measure conservation impact in terms of species' recovery through assessment of the Green Status of species [79].

### (f) RLI projections

To date, rather limited work has explored projecting future RLI trends. An RLI for terrestrial carnivores and ungulates to 2050 was estimated under different climate and land-use scenarios [80]. Extinction risk was projected to increase under a ‘business as usual’ scenario for 8%−23% of species (depending on assumptions about species’ responses to climate change). An alternative sustainable development ‘consumption change’ scenario, regardless of assumptions concerning climate change impact and dispersal, predicted an initial improvement and then stabilization in the RLI until 2030, brought about by habitat regeneration and the human-driven habitat restoration assumed for this scenario. When accounting for species' responses to climate change, this initial improvement is predicted to be followed by a decline owing to the later onset of range contractions caused by climate change, putting at risk the achievement of long-term conservation goals.

## 4. Strengths and limitations of the RLI

The RLI uses data from the IUCN Red List, so its accuracy depends on the accuracy of the underlying assessments. This may be limited by data availability, time-lags and consistency, but various factors aim to minimize these limitations. For example, the broad nature of the Red List categories (with large intervals between thresholds for different categories) means that even imprecise and inaccurate estimates of Red List parameters (e.g. population size) can accurately place species in the correct Red List category. The Red List criteria are designed to enable the utilization of a diversity of data sources so that they can be applied even to relatively poorly known species. Increasing use of internet platforms (e.g. https://forums.birdlife.org) is enabling a wide range of stakeholders to contribute information to assessments, including from more obscure publications or unpublished observations, as well as ensuring that assessments are as up-to-date as possible. Extensive guidelines [81] and a Red List Technical Working Group and Standards and Petitions Committee aim to minimize inconsistencies between assessments undertaken for different species and taxonomic groups. While some criticisms of the Red List have been published, these are often based on misconceptions and misunderstanding of the technical details (e.g. [82,83]).

The RLI is based on data for a very high proportion of species in each of the taxonomic groups included, so its trends should be largely representative of those for the entire set of species in these groups [31]. This representativeness builds from the strengths of the Red List process, which is an effective way to make meaningful inferences from imprecise or incomplete data, enabling the RLI to incorporate information even from species that are rare, localized or difficult to survey [21]. For birds at least, poorly known species with less certain estimates of population size, population trend or range size do not bias the index [31]. However, relatively few taxonomic groups (mammals, birds, amphibians, corals and cycads) currently contribute to the RLI, meaning that it is not at present representative of all biodiversity, with increased risk of bias when the index is disaggregated for small subsets of species. The breadth of taxonomic groups included will increase substantially in the next few years (see below), which will help to address this issue.

Conversely, the sensitivity of the RLI is moderately low because of the broad nature of Red List categories [68,84]. The population size, trend or distribution of species may have to change substantially before crossing the criteria thresholds to qualify for a higher or lower Red List category, and hence before changing the RLI trend. This limitation is a corollary of using the Red List categories to classify extinction risk rather than more precise quantification using population size, for example. For this reason, RLIs are complementary to population-based indices such as the Living Planet Index [85], Wild Bird Index [86] or Threatened Species Index [87]: RLIs are derived from potentially cruder data that can be collected for a higher proportion of species in a taxonomic group, while population-based indices are based on more detailed information that can often only be collected for a small (and potentially biased) subset of species.

Three other factors may contribute to time-lags between a species’ population or range size changing and this being reflected in the RLI value. First, the nature of Red List assessments means that they are mainly based on information on what has happened in the past, even though the criteria also allow integration of projected trends (e.g. criterion A3 enables species to be assessed as threatened based on projected declines if these can be justified). Second, there may be delays before changes are detected or become known to assessors. Growth in the network of thousands of scientists across the world providing detailed and up-to-date information for the Red List, improved channels of communication (e.g. https://forums.birdlife.org), rapid expansion of citizen science platforms such as eBird (https://ebird.org) and iNaturalist (https://www.inaturalist.org/), increasingly accessible machine learning (e.g. [88]), wider availability of high-resolution remote sensing (e.g. [89]) and the growth of social media (e.g. [90]) are all helping to mitigate these delays. Third, time-lags may result from long intervals between reassessments of taxa for the Red List, owing to lack of capacity. At present, funding severely constrains the rate at which species are reassessed for the Red List [91]. Nevertheless, examples of both global and national RLIs show that they can detect trends in extinction risk at policy-relevant time-scales (e.g. [63,67–71]).

The RLI and the methods for its calculation have informed the development of a number of other biodiversity indicators (e.g. [92,93]). The RLI summarizes what the Red List tells us about levels and trends in the extinction risk of species, and can be complemented with alternative metrics derived from Red List data [94]. Furthermore, extinction risk is just one dimension of the status and trends of biodiversity. It is therefore important to complement the RLI with measures of species' recovery, population abundance, ecosystem extent and condition, pressures, responses and benefits (nature’s contributions to people) to provide a more complete assessment of biodiversity loss, its drivers and consequences.

## 5. Future developments

One of the highest priorities for the future development of the RLI is to expand the breadth of taxonomic groups that are included. In the coming decade, RLIs showing trends based on comprehensive assessments will be produced for reptiles (following [95]), dragonflies (following [38]), swallowtails and conifers, as well as RLIs showing trends based on randomized samples of fishes, freshwater decapods, freshwater molluscs, orthoptera, monocots, legumes, bryophytes, pteridophytes and fungi. The aggregate RLI will be recalculated with a more recent baseline value to avoid the requirement of extrapolating trends for the newly added groups into the past. However, the resulting RLI will be considerably more representative of wider biodiversity trends, and therefore more robust as a biodiversity indicator. Different methods for aggregation across taxa (e.g. to weight species equally, or by phylogenetic diversity, etc.) could also be explored as taxonomic breadth increases. Increasing the frequency of assessments for most groups is also necessary to enable timely detection of trends. This also applies to national RLIs based on assessments of national extinction risk.

While the RLI is typically presented with confidence intervals based on various sources of uncertainty, this does not currently include the uncertainty in the underlying Red List assessments [34]. More comprehensive documentation of such uncertainty in Red List assessments is needed, in association with further development of the methods for generating confidence intervals. While the drivers of each genuine change in Red List categorization are documented, this is based on expert opinion, drawing on all available quantitative and qualitative data. Disaggregations of the RLI will be more robust if improved methods for determining the drivers can be developed and if the methods for generating such RLIs can take account of the fractional contributions of all drivers [94]. Additional potential technical developments include weighting regional and national RLIs by the proportion of the population (rather than a range) in each region or country, where relevant data are available.

Capacity-building efforts are underway to support the expansion of national Red Lists and production of national RLIs [63]. They also aim to enable regular assessment of nationally endemic species for both national red lists, the global IUCN Red List and the contribution of national data for non-endemic species to global-level assessments, therefore promoting efficient use of resources in red listing and generation of RLIs [63]. Improvements in Red List assessments (including more accurate and up-to-date parameter estimates and more detailed documentation) at both national and global scales will support the production of more robust RLIs.

Given the challenge of meeting the ambitious yet essential goals and targets in the Kunming–Montreal Global Biodiversity Framework, and the synergies and trade-offs between actions needed to meet different targets, further work to model future projections of the RLI under different policy scenarios (e.g. [79]) or land-use options would be informative. In addition, given that many developed countries significantly contribute to extinction risk beyond their borders, e.g. via the consumption of imports [96], it would be useful to explore methods to reflect such impacts abroad within national RLIs. Finally, work is underway to enhance functionality on the IUCN Red List website (https://www.iucnredlist.org/search) to enable users to undertake more sophisticated searches for different disaggregations of the RLI tailored to different end-uses, and to provide easier access to the data underpinning RLIs, including the back-cast categories and ‘genuine’ Red List category revisions. Alongside such future developments of the RLI, increased assessment of the Green Status of species (reflecting the degree to which species are ecologically functional across their indigenous range) alongside their extinction risk assessments for the IUCN Red List [79,97] will enable generation of a Green Status Index tracking trends in the recovery of species as well as the population and range size of species that are non-threatened but have depleted populations.

## 6. Conclusions

In the two decades since it was first developed, the RLI has evolved from an index of the changing fortunes of the world’s birds to an indicator that can be used to communicate levels and trends in extinction risk for a range of taxonomic groups at global, regional and national scales. It can be disaggregated by ecosystems or for relevance to different policies, and to show the impacts of different threats and efforts to mitigate these. It has consequently been widely adopted by intergovernmental conventions and environmental reporting initiatives. Despite the limitations in its taxonomic coverage and moderate sensitivity to change, the RLI has given us a broader understanding of global biodiversity trends at the species level than any other indicator. While our level of understanding of these trends is still inadequate, it is substantially greater than it was in 1992 when the CBD was signed, or in 2002 and 2012, when subequent CBD targets were agreed. To enhance its utility, increased funds are needed to strengthen the RLI through taxonomic expansion and methodological enhancements [98], and to facilitate its application in supporting the achievement of goals and targets recently adopted by the world’s governments through the Kunming–Montreal Global Biodiversity Framework. The RLI will be critical for determining whether extinction risk has been significantly reduced for known threatened species by 2030, and reduced tenfold for all species by 2050, as specified in the framework.

## Data Availability

All data for the figures presented and the raw data underpinning them are available in a Supplementary Data File. These data are kept up-to-date on the IUCN Red List website (https://www.iucnredlist.org), where graphs and data for individual RLIs are also available via the advanced search option. The R code used to calculate and plot the global RLI and its disaggregations is documented at [99]. Supplementary material is available online [100].

## Dedication

This paper is dedicated to the late Georgina Mace, who provided inspirational leadership in the development of the IUCN Red List Categories and Criteria, championed their application to an expanding set of taxa, supported the development of the Red List Index and promoted its application to track progress to global biodiversity targets.
